# Enfermedades huérfanas o raras en Colombia: situación actual y sugerencias para un futuro mejor

**DOI:** 10.7705/biomedica.8507

**Published:** 2026-06-01

**Authors:** Luis Alejandro Barrera

**Affiliations:** 1 Profesor emérito, Pontificia Universidad Javeriana Pontificia Universidad Javeriana Pontificia Universidad Javeriana Colombia; 2 Investigador emérito, Sistema Nacional de Ciencia, Tecnología e Innovación de Colombia Sistema Nacional de Ciencia, Tecnología e Innovación de Colombia Sistema Nacional de Ciencia, Tecnología e Innovación de Colombia Colombia

En Colombia, se usan las denominaciones de enfermedades huérfanas y enfermedades raras como si fueran equivalentes. En otras partes del mundo, las definiciones son distintas: enfermedades huérfanas alude a aquellas en las que no hay interés comercial en desarrollar medicamentos para ellas y, las raras, a aquellas que son muy poco frecuentes pues afectan a menos de 1 individuo por cada 2000. Según esta definición que se usa en la mayor parte del mundo, existen entre 7000 y 10 000 enfermedades raras ^1^. En Colombia, se clasifican como «raras» a las que afectan a menos de 1 individuo por cada 5000 y, actualmente, se aceptan como tales a 2273 enfermedades.

La causa en favor de las enfermedades raras comenzó a raíz de la carencia de medicamentos para su tratamiento. En 1983, en los Estados Unidos, se fundó la *National Organization for Rare Disorders* (NORD). Colombia fue el primer país de Latinoamérica en tener una ley para las enfermedades poco frecuentes: la Ley 1392 de 2010,

“[...] por medio de la cual se reconocen las enfermedades huérfanas como de especial interés y se adoptan normas tendientes a garantizar la protección social por parte del Estado colombiano a la población que padece de enfermedades huérfanas y sus cuidadores […]”.

Posteriormente, aparecieron leyes en Perú (2011), Panamá (2014), Brasil (2014) y Chile (2015), entre otros. La denominación de «enfermedad huérfana» se adoptó después de que algunos asesores de los legisladores proponentes los convencieron de que con ese calificativo se estigmatizaba menos a los pacientes.

La Ley 1392 de 2010 es de avanzada, pues incluye a los cuidadores y dispone que:

“[…] el gobierno se encargará de elaborar el listado de las enfermedades clasificadas como huérfanas que se actualizará cada dos años, establecerá guías de atención para esas enfermedades, organizará una red de centros de referencia para su diagnóstico, tratamiento y farmacia, establecerá un sistema de compra centralizada de medicamentos, financiará la investigación y organizará lo concerniente a la capacitación del talento humano y promoverá la inclusión social de los pacientes, entre otras determinaciones […]”.

Asimismo, dispuso que dichas enfermedades estarían financiadas con cargo al Seguro de Riesgos Catastróficos y Accidentes de Tránsito (ECAT) y al Fondo de Solidaridad y Garantía (FOSYGA), y facultó al gobierno para fijar condiciones y tarifas máximas de los costos de atención. En esa ley se definen las enfermedades huérfanas como aquellas con una prevalencia menor de 1 caso por cada 2000 personas. No obstante, cinco años más tarde, en la Ley Estatutaria 1751 del 2015, se cambió la definición y se estableció que para que una enfermedad se considere huérfana o rara debe tener una prevalencia menor de 1 individuo por cada 5000, con lo cual se excluyeron de esa lista a las más frecuentes.

Además, la Ley 1751 determinó que:

“[...] las personas que sufren de enfermedades huérfanas gozarán de especial protección por parte del Estado. Su atención en salud no estará limitada por ningún tipo de restricción administrativa o económica. Las instituciones que hagan parte del sector salud deberán definir procesos de atención intersectorial e interdisciplinaria que les garanticen las mejores condiciones de atención […]”.

Para tratar de que se entienda por qué nuestra legislación habla de enfermedades huérfanas cuando queremos referirnos a las raras, en los documentos oficiales recientes aparece la denominación enfermedades huérfanas y raras. Actualmente, figuran en el registro colombiano de pacientes afectados por estas enfermedades, 104 457 individuos, aunque el número total de pacientes afectados se calcula en más de dos millones. Dado que el número de pacientes identificados es poco en relación con el total posible, hasta ahora se les había podido suministrar casi todo lo prescrito, con algunos inconvenientes y demoras. Colombia era reconocido como un país donde los pacientes con enfermedades raras estaban recibiendo mejores beneficios en comparación con los países desarrollados.

Este bajo porcentaje de pacientes que están siendo beneficiados por la ley, se debe en buena medida a que los no diagnosticados viven en las áreas rurales o en la llamada «Colombia profunda», la cual constituye cerca de la mitad del país en donde no hay acceso al diagnóstico ni al tratamiento de estas enfermedades, o que, viviendo en las grandes ciudades, no están al tanto de sus derechos o no han conseguido un centro especializado que les pueda hacer el diagnóstico y peregrinan de médico en médico sin éxito.

No obstante, aun quienes estaban informados o recibían asesoría de parte de las asociaciones de pacientes, las casas comerciales, los profesionales de la salud o la defensoría del pueblo y lograron ser diagnosticados, a menudo se topaban con barreras que dilataban ese proceso hasta de 5 a 20 años, en lo que se ha llamado la «odisea diagnóstica». Las dificultades y las barreras que encontraban las familias las lograban superar especialmente mediante tutelas, lo cual originó un exagerado uso de ese recurso legal, el cual debería ser excepcional y no la forma rutinaria para reclamar el derecho a la salud.

Para hacer accesible los servicios en las regiones apartadas, en los Estados Unidos y en Europa, se han establecido redes de centros especializados que tienen recursos físicos y humanos competentes y suficientes. En Europa, estos centros atienden pacientes de los diferentes países que integran la red con la filosofía de que es mejor que el conocimiento viaje y que no lo haga el paciente.

La ley colombiana desde el 2010 preveía la creación de centros y redes, pero eso requiere, en primer lugar, que el médico que ordinariamente se ocupa de esos pacientes en los centros de atención primaria tenga la suficiente preparación para sospecharlas, incluirlas dentro de las posibilidades diagnósticas y remitir al paciente a los centros de referencia idóneos que deberían contar con procedimientos administrativos ágiles, sin trabas, para que las urgencias médicas no encuentren ninguna demora para ser atendidos. Una dificultad fundamental que se ha encontrado en la implementación de la legislación sobre estas enfermedades huérfanas y raras es que la mayor parte del personal de salud no está preparado para sospecharlas, pues hasta ahora muy poco se ha enseñado sobre ellas en las universidades.

Desde la promulgación de la ley del 2010, han surgido barreras para que la parte administrativa de los prestadores de servicios responda en forma ágil a las necesidades médicas. Sin embargo, en el 2020, algunas entidades promotoras de salud (EPS) ya estaban organizando sus propias redes y habían elaborado protocolos de atención que estaban comenzando a permitir un servicio expedito y oportuno ^2^.

En años recientes se han propuesto modificaciones en la organización, gobernanza y financiación del sistema de salud, orientadas al fortalecimiento de la atención primaria, y a la expansión y la equidad de la cobertura; sin embargo, persisten retos en la implementación de estas modificaciones, en la sostenibilidad financiera, en la disponibilidad y en el acceso oportuno a medicamentos. Estas condiciones tienen implicaciones en la atención de los pacientes, especialmente en aquellos con enfermedades huérfanas o raras, quienes presentan una mayor vulnerabilidad frente a las interrupciones de los tratamientos y las barreras de acceso.

La situación es crítica y el nuevo gobierno -cualquiera que sea su ideología o modelo de salud- tendrá que hacer correctivos urgentes: deberá dedicar suficientes recursos para entrenar al personal de la salud para tratar dichas enfermedades, organizar y articular redes de atención desde el nivel primario hasta el más especializado para que todos los pacientes con enfermedades raras, dondequiera que residan en áreas urbanas o rurales -especialmente los de sitios apartados de la geografía nacional colombiana- tengan acceso a los beneficios que les otorga la ley para su diagnóstico, tratamiento y seguimiento, en igualdad de condiciones con aquellos que viven en las cinco grandes ciudades del país, donde se concentran los recursos para atender a muchas de ellas, pero, infortunadamente, no a todas.

Esto solo será posible si se capacita a todos los profesionales de la salud en formación o en ejercicio, y se hacen campañas de divulgación masiva sobre estas enfermedades para que todos los colombianos estén enterados sobre cómo aproximarse al sistema de salud para lograr la adecuada protección, en caso de que estén afectados o les sobrevenga alguna enfermedad rara en su familia. Sin embargo, además de solucionar los apremiantes problemas mencionados, se requiere que la planeación de los servicios para las enfermedades huérfanas y raras se haga con criterio de Estado y no de gobierno, es decir que haya políticas estables y visionarias cuya continuidad esté garantizada a largo plazo.

Como es inevitable que se tenga que hacer muy pronto una reforma del sistema de salud, proponemos incluir los siguientes aspectos:

1. Apropiar urgentemente los recursos necesarios para dar a los pacientes los servicios a los que legalmente tienen derecho. Es pertinente señalar que la mayoría de las enfermedades raras son genéticas y que su cura se hace mediante terapia génica o de edición de genes, cuyo costo puede estar entre uno y tres millones y medio de dólares _^(3)^_ , y que es conveniente considerar la aparición de nuevos métodos de diagnóstico que, también, pueden ser muy onerosos. Es urgente idear mecanismos de financiación y uso óptimo de los recursos destinados a las enfermedades de alto y muy alto costo.
2. Debe ser prioritario organizar las redes y subredes para cubrir todo el país desde el nivel de atención primaria hasta los centros de referencia donde se defina el diagnóstico y se suministren los tratamientos, muchos de los cuales no están disponibles en el país y cuya importación y distribución se debe organizar para que se puedan importar y hacer llegar a donde lo requiera el paciente en pocas horas.
3. Es conveniente uniformarnos con los otros países en cuanto a la terminología. Para tratar de resolver esa confusión de las definiciones, que está creando inconvenientes para los programas y las políticas de orden transnacional sobre estas enfermedades, desde el 2022 se ha propuesto adoptar la siguiente definición:

“Una enfermedad rara es una afección médica con un patrón específico de signos, síntomas y hallazgos clínicos que afecta a menos de 1 por cada 2000 personas que viven en cualquiera de las regiones del mundo definidas por la OMS” ^4^^,^^5^.

Es conveniente que Colombia acoja esa definición para hablar un lenguaje común con la mayoría de los países del mundo ^5^^,^^6^.

Estas son algunas sugerencias para el nuevo gobierno y la nueva legislatura con el fin de avanzar y enmendar algunas determinaciones que aparecen en leyes, resoluciones y decretos. Hace más de 15 años se expidió la Ley 1392 que hemos calificado como muy buena y comprehensiva, pero su implementación, en su mayoría y en aspectos esenciales, aún está pendiente.
